# High Susceptibility to Cry1Ac and Low Resistance Allele Frequency Reduce the Risk of Resistance of *Helicoverpa armigera* to Bt Soybean in Brazil

**DOI:** 10.1371/journal.pone.0161388

**Published:** 2016-08-17

**Authors:** Patrick M. Dourado, Fabiana B. Bacalhau, Douglas Amado, Renato A. Carvalho, Samuel Martinelli, Graham P. Head, Celso Omoto

**Affiliations:** 1 Departamento de Entomologia e Acarologia, Escola Superior de Agricultura “Luiz de Queiroz”, Universidade de São Paulo, Piracicaba, São Paulo, Brazil; 2 Monsanto do Brasil Ltda, São Paulo, São Paulo, Brazil; 3 Monsanto LLC, St Louis, Missouri, United States of America; Institut Sophia Agrobiotech, FRANCE

## Abstract

The Old World bollworm, *Helicoverpa armigera* (Hübner), was recently introduced into Brazil, where it has caused extensive damage to cotton and soybean crops. MON 87701 × MON 89788 soybean, which expresses the Bt protein Cry1Ac, was recently deployed in Brazil, providing high levels of control against *H*. *armigera*. To assess the risk of resistance to the Cry1Ac protein expressed by MON 87701 × MON 89788 soybean in Brazil, we conducted studies to evaluate the baseline susceptibility of *H*. *armigera* to Cry1Ac, *in planta* efficacy including the assessment of the high-dose criterion, and the initial resistance allele frequency based on an F_2_ screen. The mean Cry1Ac lethal concentration (LC_50_) ranged from 0.11 to 1.82 μg·mL^−1^ of diet among all *H*. *armigera* field populations collected from crop seasons 2013/14 to 2014/15, which indicated about 16.5-fold variation. MON 87701 × MON 89788 soybean exhibited a high level of efficacy against *H*. *armigera* and most likely met the high dose criterion against this target species in leaf tissue dilution bioassays up to 50 times. A total of 212 F_2_ family lines of *H*. *armigera* were established from field collections sampled from seven locations across Brazil and were screened for the presence of MON 87701 × MON 89788 soybean resistance alleles. None of the 212 families survived on MON 87701 × MON 89788 soybean leaf tissue (estimated allele frequency = 0.0011). The responses of *H*. *armigera* to Cry1Ac protein, high susceptibility to MON 87701 × MON 89788 soybean, and low frequency of resistance alleles across the main soybean-producing regions support the assumptions of a high-dose/refuge strategy. However, maintenance of reasonable compliance with the refuge recommendation will be essential to delay the evolution of resistance in *H*. *armigera* to MON 87701 × MON 89788 soybean in Brazil.

## Introduction

*Helicoverpa armigera* (Hübner) is considered to be one of the most important agricultural pests in the world [1]. This polyphagous species is widespread throughout Europe, Africa, Asia, and Australia, where it causes extensive damage to a wide range of crops [2–4]. The ability of *H*. *armigera* to persist in agricultural areas and adapt to changes in farming practices is one of the major factors contributing to the pest status of this species [5].

In the Old World and in Australia, where *H*. *armigera* is a major pest of cotton, this species is the primary target of genetically modified cotton containing insecticidal proteins from *Bacillus thuringiensis* (Bt), which has been widely adopted by growers to overcome insecticide resistance issues [6–8]. Recently this species was identified in South America, especially in Brazil [9, 10]. Several studies also indicated the risk of a successful invasion of this species into North America [11, 12] and the Animal and Plant Health Inspection Service of the United States Department of Agriculture (USDA-APHIS) reported detection of *H*. *armigera* in Puerto Rico in 2014 [13] and in the continental United States in 2015 [14]. In Brazil, *H*. *armigera* has been detected on a variety of crops, particularly dicots and to lesser extent monocots [15, 16]. Among these host crops, soybean and cotton represent the largest areas in Brazil, totaling 30 million and 1 million hectares per year, respectively [1].

In different parts of the world, *H*. *armigera* represents a significant insect management challenge to cotton and soybean growers [6, 17–19]. Likewise, the widespread distribution of *H*. *armigera* in Brazil was identified as a risk to the effective and sustainable use of Bt crops in South America. Bt cotton has been widely used with success in many countries including Brazil [20, 21] where a Bt soybean product expressing the protein Cry1Ac (MON 87701 × MON 89788) was recently approved for cultivation [22] and commercially launched in crop season 2013/14. The high efficacy of MON 87701 × MON 89788 soybean against major lepidopteran pests demonstrated its potential to become part of an Integrated Pest Management (IPM) program aimed at reducing insecticide use [23, 24–25]. Although soybean looper, *Chrysodeixis includens* (Walker), stands out as the predominant soybean pest in Brazil, *H*. *armigera* has recently become more prevalent in Brazilian soybean-growing areas [26] and the risk of resistance evolution in this species to Bt soybean must be considered in Insect Resistance Management (IRM) programs.

As anticipated, MON 87701 × MON 89788 soybean has been rapidly adopted by growers in Brazil, likely due to the high levels of control provided against the main lepidopteran pests, especially *C*. *includens*, *Anticarsia gemmatalis* Hübner, *Chloridea virescens* (F.) and *H*. *armigera* [23, 24, 27–29]. As with other Bt crops, the primary threat to the sustainable use of MON 87701 × MON 89788 soybean is the evolution of resistance by target pests [30]. The most effective strategy for managing insect resistance to Bt crops, the high-dose/refuge strategy, is based on the assumptions that resistance alleles to a Bt protein are rare; that a Bt protein is consistently produced by a plant at a highly toxic concentration, reducing the selective differential between susceptible (SS) and heterozygous (RS) target insects, therefore making resistance alleles “functionally recessive”; and that refuge areas with non-Bt plants are cultivated to allow the development of susceptible (i.e., unselected) insects [30, 31]. Three key factors are consistently associated with the cases of field-evolved resistance reported thus far [32–37]: the deployment of single-mode-of-action Bt products, failure to meet the high-dose criterion, and poor refuge compliance [31, 38].

Previous studies reported high toxicity of MON 87701 × MON 89788 soybean against *H*. *armigera* [28, 29]. In this paper, we describe baseline susceptibility curves to Cry1Ac of several field *H*. *armigera* populations sampled from soybean and cotton fields and present data regarding toxicological attributes of MON 87701 × MON 89788 soybean against *H*. *armigera* generated in the laboratory and screenhouse, including a high-dose assessment. Furthermore, we present results of an F_2_ screen [39] for rare Cry1Ac resistance alleles carried out using Bt soybean leaf tissue to improve the sensitivity of estimates of resistance allele frequencies. Finally, we discuss the IRM implications of having large areas in Brazil cultivated with Bt technologies targeting *H*. *armigera*.

## Results

### Baseline susceptibility to Cry1Ac

The baseline susceptibility of *H*. *armigera* documented using a diet-incorporated bioassay for insects sampled in crop seasons 2013/14 and 2014/15 indicated neonate larvae were highly susceptible to Cry1Ac with LC_50_ values (i.e., concentrations that caused 50% mortality of *H*. *armigera* larvae) ranging from 0.11 (populations MS-1 and SP-1) to 1.82 μg Cry1Ac mL^−1^ diet (population BA-2) (Table 1) and EC_50_ values (i.e., concentrations that caused 50% growth inhibition) ranging from 0.0029 (population MS-1) to 0.0165 μg Cry1Ac mL^−1^ diet (population BA-4) (Table 2). There were overlapping responses (LC_50_ and EC_50_) and relative low variability across populations; the most susceptible and most tolerant *H*. *armigera* populations differed by approximately 16.5-fold and 5.7-fold for the LC_50_ and EC_50_ values, respectively.

**Table 1:** Summary of concentration-mortality (lethal concentration [LC]) of *H*. *armigera* neonates exposed to Cry1Ac protein incorporated into artificial diet.

Season^a^	Population	Generation	n^b^	Slope ± SE	LC_50_ (μg mL^−1^ diet)^c^		LC_90_ (μg mL^−1^ diet)^c^		Goodness of fit	
					Concentration	95% CI	Concentration	95% CI	χ^2^	df^d^
**2013/14**	**LAB**	**F**_**4**_	**688**	**1.59 ± 0.13**	**1.22**	**0.92–1.56**	**7.83**	**5.88–11.1**	**0.79**	**5**
	**MS-1**	**F**_**3**_	**896**	**1.94 ± 0.13**	**0.11**	**0.08–0.13**	**1.43**	**1.02–2.15**	**6.79**	**5**
	**SP-1**	**F**_**2**_	**896**	**1.53 ± 0.10**	**0.11**	**0.08–0.14**	**3.15**	**2.07–5.29**	**7.84**	**5**
	**BA-1**	**F**_**2**_	**896**	**2.00 ± 0.13**	**1.37**	**0.95–1.81**	**5.99**	**4.55–8.56**	**4.94**	**5**
	**PR-1**	**F**_**2**_	**899**	**1.89 ± 0.12**	**0.12**	**0.09–0.15**	**1.76**	**1.25–2.67**	**3.88**	**5**
	**MT-1**	**F**_**2**_	**896**	**1.64 ± 0.17**	**0.41**	**0.32–0.52**	**8.96**	**5.90–15.0**	**2.20**	**5**
	**GO-1**	**F**_**2**_	**896**	**2.04 ± 0.13**	**0.19**	**0.15–0.22**	**2.22**	**1.60–3.28**	**5.21**	**5**
	**PR-2**	**F**_**2**_	**896**	**2.17 ± 0.18**	**0.32**	**0.26–0.39**	**3.35**	**2.44–4.90**	**4.15**	**5**
	**BA-2**	**F**_**2**_	**1152**	**2.11 ± 0.24**	**1.82**	**1.16–2.49**	**7.89**	**5.71–12.5**	**3.46**	**4**
	**BA-3**	**F**_**3**_	**720**	**2.74 ± 0.11**	**0.32**	**0.26–0.39**	**3.35**	**2.44–4.90**	**1.54**	**5**
**2014/15**	**MT-2**	**F**_**2**_	**896**	**1.50 ± 0.12**	**0.20**	**0.22–0.36**	**1.66**	**1.36–2.02**	**3.45**	**5**
	**SP-2**	**F**_**2**_	**896**	**1.87 ± 0.13**	**0.40**	**0.36–0.59**	**0.60**	**0.43–0.91**	**1.43**	**5**
	**MT-3**	**F**_**2**_	**896**	**1.66 ± 0.13**	**0.28**	**0.18–0.48**	**1.28**	**0.56–1.64**	**1.07**	**5**
	**BA-4**	**F**_**2**_	**768**	**1.90 ± 0.13**	**0.44**	**0.35–0.55**	**15.35**	**9.13–30.1**	**1.27**	**5**
	**PR-3**	**F**_**2**_	**896**	**1.43 ± 0.09**	**0.32**	**0.25–0.41**	**10.76**	**6.69–19.6**	**4.79**	**5**

^a^ Season when populations were collected.

^b^ Number of individuals tested.

^c^ LC_50_ and LC_90_ are the concentrations of Cry1Ac protein (μg mL^−1^ diet) that cause death or inhibit molting beyond first instar of 50% and 90% of individuals, respectively, after 7 days of bioassay. CI, confidence interval.

^d^ Degrees of freedom.

**Table 2:** Summary of effective concentration (EC, or growth inhibition concentration) of *H*. *armigera* neonates exposed to Cry1Ac protein incorporated into artificial diet.

Season^a^	Population	Generation	n^b^	EC_50_ (μg mL^−1^ diet)^c^		EC_90_ (μg mL^−1^ diet)^c^	
				Concentration	95% CI	Concentration	95% CI
**2013/14**	**LAB**	**F**_**4**_	**523**	**0.0028**	**0.0018–0.0049**	**0.0189**	**0.0062–0.0249**
	**MS-1**	**F**_**3**_	**447**	**0.0029**	**0.0021–0.0037**	**0.0421**	**0.0344–0.0526**
	**SP-1**	**F**_**2**_	**466**	**0.0034**	**0.0031–0.0038**	**0.0407**	**0.0374–0.0444**
	**BA-1**	**F**_**2**_	**468**	**0.0072**	**0.0038–0.0082**	**0.0468**	**0.0422–0.2120**
	**PR-1**	**F**_**2**_	**451**	**0.0055**	**0.0049–0.0060**	**0.0341**	**0.0301–0.0389**
	**MT-1**	**F**_**2**_	**593**	**0.0079**	**0.0038–0.0128**	**0.1848**	**0.0894–0.4885**
	**GO-1**	**F**_**2**_	**507**	**0.0036**	**0.0020–0.0052**	**0.0547**	**0.0371–0.0871**
	**PR-2**	**F**_**2**_	**563**	**0.0033**	**0.0024–0.0042**	**0.0658**	**0.0261–0.0842**
	**BA-2**	**F**_**2**_	**478**	**0.0032**	**0.0018–0.0050**	**0.0602**	**0.0274–0.0644**
	**BA-3**	**F**_**3**_	**436**	**0.0050**	**0.0041–0.0059**	**0.0554**	**0.0452–0.0695**
**2014/15**	**MT-2**	**F**_**2**_	**307**	**0.0032**	**0.0002–0.0040**	**0.0581**	**0.0470–0.0738**
	**SP-2**	**F**_**2**_	**353**	**0.0030**	**0.0026–0.0038**	**0.0590**	**0.0500–0.0708**
	**MT-3**	**F**_**2**_	**316**	**0.0032**	**0.0002–0.0038**	**0.0590**	**0.0501–0.0708**
	**BA-4**	**F**_**2**_	**454**	**0.0165**	**0.0128–0.0209**	**0.2724**	**0.1676–0.4939**
	**PR-3**	**F**_**2**_	**418**	**0.0160**	**0.0153–0.0168**	**0.1143**	**0.1030–0.1275**

^a^ Season when populations were collected.

^b^ Number of individuals tested.

^c^ EC_50_ and EC_90_ are the effective concentrations of protein required to cause 50% and 90% growth inhibition, respectively, at 7 days. CI, confidence interval.

### Efficacy trials—Leaf disc and screenhouse

**Fig 1:**
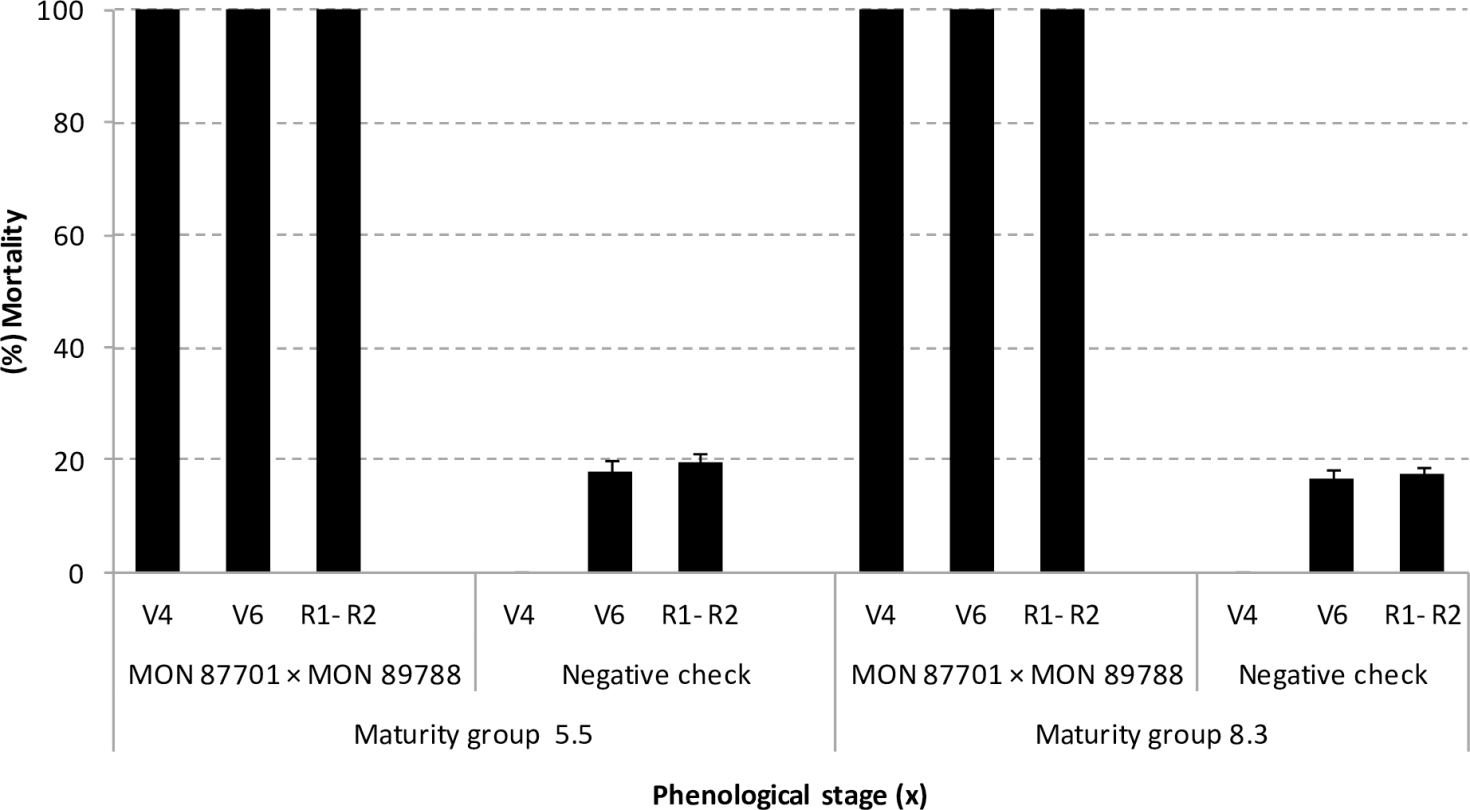
*H*. *armigera* mortality on leaf discs of MON 87701 × MON 89788 and near isogenic negative checkschecks soybeans at different phenological stages. The mean percentage of mortality of *H*. *armigera* neonates was assessed after 5 days on leaf discs of MON 87701 × MON 89788 soybean and near-isogenic negative checks of maturity groups 5.5 and 8.3. There were statistically significant differences (*t*-test, *P* ≤ 0.05) between MON 87701 × MON 89788 soybean and near-isogenic negative checks at all phenological stages evaluated (df = 10; *P* < 0.0001).

The Cry1Ac protein expressed in leaves of MON 87701 × MON 89788 soybean of maturity groups 5.5 and 8.3 was highly toxic to *H*. *armigera* neonates in all phenological stages evaluated, resulting in 100% mortality after five days of leaf disc bioassay (Fig 1).

Evaluation in screenhouse with intense artificial infestation has shown that no *H*. *armigera* larvae were able to establish on MON 87701 × MON 89788 soybean plants, in contrast to a large number of larvae per meter observed on negative checks (Table 3). Reductions in damage on MON 87701 × MON 89788 soybean were observed as a consequence of *H*. *armigera* larvae control. Thirty-five days after infestation, accumulated defoliation reached the maximum defined level (50% defoliation) on negative checks, significantly higher than on MON 87701 × MON 89788 soybean of either maturity group tested.

**Table 3:** Larval incidence and plant damage on MON 87701 × MON 89788 and control soybean under high-pressure *H*. *armigera* infestation in screenhouse trials.

Material	Larvae per meter	Defoliation (%)	Pods/plant	Pods damaged (%)
	**Maturity group 5.5**			
**MON 87701 × MON 89788**	0.0 ± 0.0*	0.0 ± 0.0*	**30.0 ± 1.1**	0.0 ± 0.0*
**Negative checks**	**480.6 ± 32.2**	**50.0 ± 0.0**	**37.5 ± 1.0**	**37.2 ± 1.9**
	**Maturity group 8.3**			
**MON 87701 × MON 89788**	0.0 ± 0.0*	0.0 ± 0.0*	72.6 ± 4.1*	0.0 ± 0.0*
**Negative checks**	**463.3 ± 25.9**	**50.0 ± 0.0**	**0.7 ± 0.2**	**94.6 ± 11.2**

Values of Larval incidence were measured as larvae per meter row. Damage was measured on vegetative (percentage defoliation) and reproductive structures (pods per plant and pods damaged). For each measurement, MON 87701 × MON 89788 soybean of maturity groups 5.5 and 8.3 was compared to non-Bt checks of the same maturity groups.

* There were statistically significant differences (*t*-test; *P* ≤ 0.05) between MON 87701 × MON 89788 soybean and negative checks of both maturity groups for larvae per meter, defoliation, and pods damaged, and in maturity group 8.3 for pods/plant (df = 6, *P* < 0.0001).

In addition to its effects on vegetative tissues, *H*. *armigera* has podworm behavior and can damage reproductive structures including flowers and pods. In the case of non-Bt soybean with a longer life cycle (maturity group 8.3), the plants were starting to flower at the time that *H*. *armigera* eggs hatched. *H*. *armigera* larvae fed on these flowers and consequently reduced the number of pods developed on negative check plants by ~99% relative to that of MON 87701 × MON 89788 soybean in the same maturity group (df = 6, *P* < 0.0001; Table 3). In contrast, the number of pods on non-Bt soybean with a shorter life cycle (maturity group 5.5) was not significantly different from that of MON 87701 × MON 89788 soybean of the same maturity group (df = 6, *P* = 0.066). However, for both maturity groups tested, the developed pods of MON 87701 × MON 89788 soybean were significantly less damaged than those of the corresponding negative checks.

### High-dose assessment

**Fig 2:**
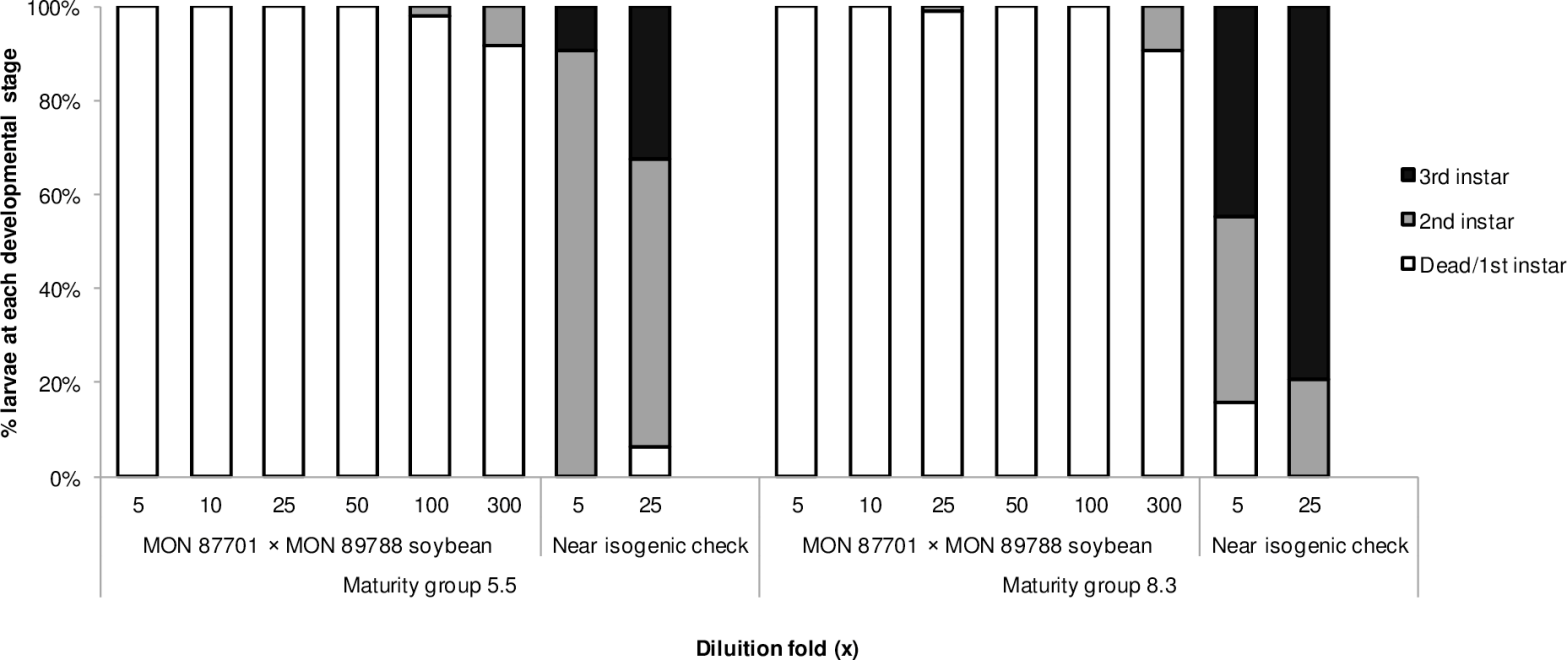
*H*. *armigera* development on diet containing MON 87701 × MON 89788 or control soybean leaf. Mortality and larval development of *H*. *armigera* neonates was assessed after 7 days of feeding on artificial diet containing lyophilized leaf tissue at serial dilutions of 5- to 300-fold relative to fresh leaf tissue for MON 87701 × MON 89788 and 5- to 25-fold relative to the fresh tissue for the near-isogenic negative checks. Bars indicate mean percentages of dead or first-instar larvae (% mortality) and of second- and third-instar larvae.

As described previously for other Bt soybean target pests such as *C*. *includens*, *A*. *gemmatalis* and *C*. *virescens* [23, 24], lyophilized leaf tissue of MON 87701 × MON 89788 soybean of maturity groups 5.5 and 8.3 caused high mortality (larvae dead or not molting beyond first instar) and stunting (larvae not molting to third instar) of *H*. *armigera* when diluted up to 25-fold in artificial diet (Fig 2). In contrast, near isogenic negative checks tissue diluted 5- and 25-fold in artificial diet caused low levels of mortality, ranging from 6.25% to 15.62% across the two maturity groups. Overall, most of the larvae exposed to artificial diet containing non-Bt soybean tissue reached the second instar after seven days, while most of the larvae on a diet containing lyophilized tissue of MON 87701 × MON 89788 soybean did not survive or develop past the first instar.

### Frequency of resistance alleles to MON 87701 × MON 89788 soybean

Of the 870 single pairs of *H*. *armigera* moths obtained from larvae collected from crop production field, only 212 (24%) successfully progressed to the stage where F_2_ neonate larvae were exposed to MON 87701 × MON 89788 soybean leaf tissue expressing the Cry1Ac protein (Table 4). A total of 212 F_2_ two-parent lines (originating from 424 feral individuals) of *H*. *armigera* were screened for resistance to Cry1Ac using fresh leaf tissue of MON 87701 × MON 89788 soybean. Among these lines, no surviving *H*. *armigera* larvae were found after four days, indicating that none of the 212 families carried major resistance alleles to Cry1Ac. The estimated resistant allele frequency was 0.0011 (95% confidence interval of 0–0.0043) (Table 4).

**Table 4:** Initial frequency of MON 87701 × MON 89788 soybean resistance alleles in *H*. *armigera* from Brazil.

Population code	Single pairs (F_0_)	F_1_parental isoline families	F_2_ parental isoline families		Estimated R frequency (95% CI)^a^
			Screened	Positive	
**BA-1**	**127**	**99**	**11**	**0**	**0.0194 (0–0.0701)**
**BA-2**	**145**	**120**	**51**	**0**	**0.0047 (0–0.0174)**
**BA-3**	**64**	**85**	**37**	**0**	**0.0064 (0–0.0236)**
**BA-4**	**120**	**100**	**37**	**0**	**0.0064 (0–0.0236)**
**MT-1**	**83**	**100**	**12**	**0**	**0.0180 (0–0.0652)**
**MT-2**	**104**	**88**	**28**	**0**	**0.0084 (0–0.0306)**
**SP-2**	**81**	**42**	**36**	**0**	**0.0066 (0–0.0242)**
**Total**	**870**	**684**	**212**	**0**	**0.0011 (0–0.0043)**

^a^ Frequency estimates for each population and for the pooled data (Total). CI, confidence interval.

## Discussion

The overlap in the LC_50_ and EC_50_ values obtained for populations from different regions in Brazil indicated low intra-specific variability (Table 1) [40]. Although *H*. *armigera* populations were sampled across diverse regions, recent invasions and geographic expansion coupled with high mobility may sustain low levels of intra-specific variability in Brazil [16]. In contrast, variation in susceptibility to Cry1Ac of up to 50-fold previously has been described across different world areas and may be associated with natural variation in heliothine pest populations and with several other factors that may affect the outcome of bioassays, such as the source of Cry1Ac protein and the bioassay technique [41–44].

The responses of *H*. *armigera* populations sampled across Brazil are comparable to those obtained with a Cry1Ac (MVPII) diet-incorporation bioassay from cotton-growing areas in India two years prior to the commercial launch of Bt cotton. In those tests, the LC_50_ ranged between 0.11 and 0.61 μg mL^−1^ diet and the LC_90_ ranged between 1.17 and 6.94 μg mL^−1^ diet [45]. Susceptibility of *H*. *armigera* to Cry1Ac protein in Brazil also was comparable to the Cry1Ac susceptibility of *C*. *includens* and *C*. *virescens*, both additional key target pests of MON 87701× MON 89788 soybean [23, 24, 46].

Due to the importance of the Cry1Ac protein for Bt cotton globally, susceptibility of *H*. *armigera* to Cry1Ac has been well documented through baseline susceptibility studies in China, India, Australia and Africa [43, 47–49]. Nevertheless, establish in regional susceptibility baselines allows to detect shifts in susceptibility due to the evolution of resistance [50]. Resistance monitoring programs using phenotypic methods consist of collecting insect populations in the field and exposing their offspring to diagnostic concentrations established using baseline susceptibility data [51, 52]. The use of diagnostic concentrations to monitor resistance has the advantage of being more efficient for detecting low frequencies of resistance because all individuals are tested at an appropriate concentration, requiring fewer individuals and less time than complete concentration-mortality tests, and enabling a greater number of populations to be tested [51, 53]. Despite of the limitations of this method, such as limited sensitivity to detect recessive resistant alleles, this type of bioassay is compatible with a large-scale procedure for monitoring the susceptibility of MON 87701× MON 89788 soybean target pests [24, 41]. The response criterion that was used herein represented not only mortality but also larval stunting (i.e., first-instar larvae were considered dead). The use of this response criterion more accurately represents field responses and produces more appropriate diagnostic concentrations that can detect small changes in population susceptibility [54] and makes the monitoring of susceptibility faster and more practical [55]. The results presented in this paper provide a robust base for establishing and validating diagnostic concentrations to be used in a program to monitor the susceptibility of *H*. *armigera* populations to Cry1Ac in Brazil.

Major factors associated with the sustained usefulness of Bt crops are a high concentration of the Bt protein, causing practically complete mortality (e.g., ≥99.99%) of an insect pest at the field level and thus rendering inheritance of resistance to be functionally recessive; a low initial frequency of resistance alleles; and abundant refuges of non-Bt host plants available in proximity to the Bt crop [38, 56]. Several studies have reported the expected efficacy of MON 87701 × MON 89788 against the main soybean pests in South America, such as *A*. *gemmatalis*, *C*. *includens*, another soybean looper (*Rachiplusia nu*), the soybean shoot borer (*Crocidosema aporema* [Walsingham, 1914]), and *C*. *virescens* [23, 24, 27]. High efficacy of MON 87701 × MON 89788 against *H*. *armigera* was reported by Azambuja et al. [29], who documented 100% mortality of later-instar *H*. *armigera* larvae feeding on MON 87701 × MON 89788 soybean leaf tissue. Effective protection of MON 87701 × MON 89788 soybean against *H*. *armigera* was also reported by Yu et al. [28]; however, these authors measured survival of 2^nd^-instar larvae and larval weight only after four days of feeding on the tested material, whereas Azambuja et al. [29] infested with neonate larvae and checked for larval mortality up to six days. In the latter case, all larvae fed on MON 87701 × MON 89788 soybean leaf tissue died four to six days after infestation. The most direct approach to test the high-dose assumption is to allow resistant and susceptible adults to mate in the laboratory and measure the survival of their hybrid progeny on Bt plants. However, given that suitable resistant strains for direct tests are not available, indirect tests were used [56]. High mortality and stunting of *H*. *armigera* obtained with a range of leaf tissue dilutions (5 to 100x) indicated that the expression of Cry1Ac protein in MON 87701 × MON 89788 soybean should be capable of controlling most heterozygous resistant insects (i.e., individuals carrying one copy of the resistance allele), causing resistance to be functionally recessive [30, 38, 56–58].

Results obtained from the series of experiments presented herein demonstrated the high efficacy of MON 87701 × MON 89788 soybean against *H*. *armigera*. The high efficacy against *H*. *armigera* reflects both the relative susceptibility of *H*. *armigera* to Cry1Ac and very high Cry1Ac expression levels. The Cry1Ac protein expressed in MON 87701 × MON 89788 is identical to the Bt protein expressed in MON 531 cotton (Bollgard) and MON 15985 cotton (Bollgard II), which display virtually complete efficacy against tobacco budworm (*C*. *virescens*) and pink bollworm (*Pectinophora gossypiella* [Saunders]) and moderate activity against *Helicoverpa zea* (Boddie) and *H*. *armigera* [59,60]. However, the Cry1Ac expression levels in leaves of Bt cotton are significantly lower (around 5μg/g dry weight) [61] than the levels found in MON 87701 × MON 89788 soybean (from 50 to 200 μg/g fresh weight) [27].

The initial frequency of resistance alleles estimated herein (0.0011 (95% confidence interval of 0–0.0043)) overlaps with values obtained in Cry1Ac resistance monitoring programs in Australia using F_2_ screening over seven years for *H*. *armigera* (0.0006 (95% confidence interval of 0.0001–0.002)) [17]. In Brazil, a comparable Cry1Ac resistance allele frequency (0.0004) was recently estimated for *C*. *includens* using the same methods [46]. Although the F_2_ screen method provides sensitive estimates of resistance allele frequency in field populations, it requires more efforts than the use of diagnostic concentrations when used as tool in a routine resistance monitoring program [39]. The high toxicity of Bt soybean against *H*. *armigera* and the low estimated frequency of resistance allele meet the requirements for the high-dose/refuge IRM strategy [30, 38, 57].

The other critical element of the high-dose refuge strategy is that sufficient refuge exists in the form of plants that can serve as hosts for the pests but do not produce Bt proteins to allow the survival of susceptible insects [30]. *H*. *armigera* is a highly polyphagous species that develops on many cultivated and non-cultivated plants. In China, for instance, refuges of non-Bt cotton have not been required for Bt cotton that produces Cry1Ac [8, 62, 63]. The approach implemented in China was based on the premise that abundant non-Bt host plants of *H*. *armigera* in China would provide sufficient natural refuges to delay the evolution of resistance. Although several studies have reported small shifts in the frequency of *H*. *armigera* resistance to Cry1Ac, Bt cotton producing Cry1Ac has continued to provide substantial control of this pest in China [63]. In addition to the availability of a high proportion of non-Bt host plants, the small size of farms in China coupled with the high mobility of *H*. *armigera* are typically pointed out as factors contributing to the success of the natural refuge strategy for *H*. *armigera* in China [8, 64, 65].However, recent modeling suggests that natural refuges delayed resistance to Cry1Ac in *H*. *armigera* in China but may not have been as effective as an equivalent area of non-Bt cotton refuge, and that switching to Bt cotton producing two or more Bt proteins and integrating other control tactics could slow further resistance evolution [20].

The agricultural landscape in Brazil poses additional challenges for managing resistance to Bt crops. Warmer temperatures and longer growing seasons than in temperate regions allow for a build-up of insect pest populations, particularly polyphagous species [21]. Soybean cultivation is characterized by economies of scale and despite variation in farm size across the landscape, the average farm in the important Center-West soybean-producing region is six times the Brazilian average [66]. Bt proteins are available in Brazil in three main row crops that can host *H*. *armigera* (soybean, cotton, and maize), creating a cross-crop scenario whose outcome depends on the ecological interaction between the pest and its host plants and the efficacy of the different Bt crops in the system. There is no information on the spatial and temporal variability of host plant use by *H*. *armigera* in Brazil. These data could improve help in the design of IRM programs for Brazil [67]. MON 87701 × MON 89788 soybean was recently approved for cultivation in Brazil [22] and has been rapidly adopted by growers due to the high levels of control provided against the main lepidopteran pests of soybean [68]. Recognizing the risk of resistance evolution in this challenging landscape, growers in Brazil are recommended to plant and manage a structured refuge of non-Bt soybean equivalent to at least 20% of the total soybean area being cultivated. Seed mixtures are not recommended because studies indicated that no target pests survive on non-Bt plants interspersed in a Bt soy field (data not published). Maintenance of sufficient compliance with this structured refuge recommendation at the farm level is essential to delay the evolution of resistance in *H*. *armigera* and other target pests of MON 87701 × MON 89788 soybean. The planting of structured refuges for Bt crops is not mandatory in Brazil, thus engaging key stakeholders across the soybean production chain is critical to effectively reach growers and ensure facilitate the planting of refuges. In addition to effective implementation of refuges, the deployment of technologies pyramiding insect-protection traits is a strategy capable of slowing the evolution of insect resistance, and this approach should be pursued in Brazil.

## Materials and Methods

### Insect rearing and field collections

Permit access to collect material used in our research at various crop sites was granted by Sistema de Autorização e Informação em Biodiversidade (Sisbio) from the Brazilian Ministry of Environment to SGS Gravena (Sisbio License # 10018–1) and PROMIP (Sisbio License # 40380–2). Number of caterpillars collected and location are listed in Table 5.The insect colony used for characterizing the toxicological parameters of MON 87701 × MON 89788 soybean against *H*. *armigera* (leaf disc, screenhouse and dilution bioassays) was sampled on soybean in Goiás State during crop season 2013/14 and established in the laboratory as a reference population (LAB). Field populations were sampled on non-Bt soybean and non-Bt cotton from crop season 2013/14 to 2014/15 from multiple regions across Brazil to establish Cry1Ac baseline susceptibility and to perform an F_2_ screen (Table 5). Approximately 800–1,200 larvae were collected in each sampling area and kept on an artificial diet adapted from Kasten et al. [69]. Mortality caused mainly by parasitism and entomopathogens resulted in approximately 40% adult viability. Remaining adults were maintained in oviposition cages. To guarantee the absence of *H*. *zea* individuals in the populations, all adult males (F_0_) of each population were captured after mating and their genitalia were dissected and identified [2]. Populations were not used unless all individuals sampled were identified as *H*. *armigera*.

**Table 5:** Locations, host plant and crop season of *H*. *armigera* field populations sampled.

Season	Code	County/State	Host	Latitude	Longitude
	**LAB**	**Montividiu, GO**	**Soybean**	**17°22'28.8"S**	**51°13'23.3"W**
	**MS-1**	**Chapadão do Sul, MS**	**Soybean**	**18°44'38.5"S**	**52°35'17.8"W**
	**SP-1**	**Jaboticabal, SP**	**Soybean**	**21°12'27.8"S**	**48°19'37.0"W**
	**BA-1**	**Luis Eduardo Magalhães, BA**	**Cotton**	**12°00'56.9"S**	**45°49'19.8"W**
	**PR-1**	**Ponta Grossa, PR**	**Soybean**	**25°05'13.5"S**	**49°59'18.7"W**
**2013/14**	**MT-1**	**Primavera do Leste, MT**	**Soybean**	**15°26'22.4"S**	**54°10'44.7"W**
	**GO-1**	**Rio Verde, GO**	**Soybean**	**17°44'27.0"S**	**50°55'55.4"W**
	**PR-2**	**Rolândia, PR**	**Soybean**	**23°14'34.9"S**	**51°26'14.7"W**
	**BA-2**	**Correntina, BA**	**Soybean**	**13°21'57.6"S**	**45°28'36.2"W**
	**BA-3**	**Luis Eduardo Magalhães, BA**	**Soybean**	**11°48'26.6"S**	**45°59'01.3"W**
	**MT-2**	**Primavera do Leste, MT**	**Cotton**	**15°20'18.6"S**	**54°20'23.0"W**
	**SP-2**	**Jaboticabal, SP**	**Soybean**	**21°12'50.7"S**	**48°15'58.8"W**
**2014/15**	**MT-3**	**Lucas do Rio Verde, MT**	**Soybean**	**12°58'15.7"S**	**56°08'20.9"W**
	**BA-4**	**Luis Eduardo Magalhães, BA**	**Soybean**	**12°11'27.9"S**	**45°46'28.5"W**
	**PR-3**	**Londrina, PR**	**Soybean**	**23°26'38.2"S**	**51°06'04.8"W**

### Baseline susceptibility to Cry1Ac

A synthetic Cry1Ac protein formulated product (MVP II, *Pseudomonas* encapsulated Cry1Ac from Dow Chemicals, San Diego, CA, USA, containing 11.14% of active Cry1Ac protein) was incorporated into the artificial diet, without formalin and antibiotics, when the diet temperature reached 56°C. To establish the baseline curves, five to seven concentrations were used, ranging from 0.01 to 10 μg of active protein ml^−1^ of diet. A 1-ml aliquot of diet containing the protein was poured into each cell of a 16-cell square area of a 128-cell bioassay tray. The trays were sealed with self-adhesive plastic sheets (BIO-CV-16; CD International Inc., Pitman, NJ, USA) that allowed gas exchange with the external environment and then placed in a climatic chamber (temperature 27 ± 2°C, 60 ± 10% relative humidity, and a 14-h photoperiod). The experimental design was completely randomized, with seven to eight replicates per concentration (16 larvae per replicate). Mortality (individuals dead and inhibited from molting beyond first instar) and the weight of the surviving larvae were recorded after seven days to estimate two toxicological parameters: lethal concentration (LC) and effective concentration (EC) [70]. Toxicological parameters LC_50_, LC_90_, EC_50_, and EC_90_ and the respective confidence intervals (CI 95%) were estimated using JMP® v10.0 (SAS Institute Inc, Cary, NC, USA).

### Efficacy trials—Leaf disc and screenhouse

Efficacy of MON 87701 × MON 89788 soybean plants against *H*. *armigera* was assessed by using leaf disc and screenhouse trials as previously described [23, 24]. MON 87701 × MON 89788 soybean and near isogenic negative checks of maturity groups 5.5 and 8.3 (different rates of growth and development) were grown in a greenhouse. Completely expanded leaves were removed from the upper third of the plants when they reached phenological stages V4, V6, and R1–R2. Leaf discs 1.2 cm in diameter were cut using a metallic cutter and placed on mixture of water–agar at 2.5% (1 mL cell−1) separated by a filter paper disc in 24-well acrylic plates (Costar®; Corning, Tewksbury, MA, USA). The experimental design was completely randomized with five replicates per treatment (24 neonates per replicate). Larval mortality was recorded at five days after infestation.

For the screenhouse trials, 5,000 *H*. *armigera* pupae were used to infest a containment system consisting of nylon net cages (16×18 mesh; 13.0 m length × 3.5 m width × 2.9 m height) when plants reached the R1 reproductive stage. Evaluations of larval incidence, defoliation, and damaged pods were taken at 32 days after adult emergence.

JMP® v10.0 (SAS Institute Inc, Cary, NC, USA) was used to perform statistical analysis. Data were transformed prior to statistical analysis to meet the assumption of normality. Mortality data obtained from laboratory bioassays (*x*) were transformed into $\left(\sqrt{x+0.5}\right)$ and screenhouse data (*x*) were transformed into (*x* + 0.1)^0.5^. Statistical evaluation was performed using Student's *t*-test.

### High-dose assessment

To assess the high-dose concept for MON 87701 × MON 89788 soybean against *H*. *armigera*, lyophilized and macerated leaf tissue at several dilutions was incorporated into artificial diet as previously described [23, 24]. To produce tissue, MON 87701 × MON 89788 soybean and near isogenic negative checks of maturity groups 5.5 and 8.3 were grown in a greenhouse. Leaves from the upper canopy were removed from MON 87701 × MON 89788 soybean and near isogenic negative checks plants at phenological stage R1–R2 and macerated to be incorporated into artificial diet. Dilutions tested in the diet bioassays ranged from 5- to 300-fold relative to the fresh tissue for MON 87701 × MON 89788 and 5- to 25-fold relative to the fresh tissue for the near isogenic negative checks controls. Bioassay and evaluation procedures were performed as described previously for baseline susceptibility.

### Frequency of resistance alleles to MON 87701 × MON 89788 soybean

The F_2_ screen was conducted according to Huang et al. [71]. Pupae of *H*. *armigera* derived from individuals sampled on non-Bt soybean and non-Bt cotton plants were sexed and individually placed in cylindrical plastic cages (20 cm height × 10 cm diameter) until emergence. Adults were matched and allowed to mate and oviposit. Each single-pair mating represented an insect family line. Approximately 100 F_1_ progeny larvae of each single-pair mating were then reared on artificial diet to the pupal stage, as described above. F_1_ adults (30 couples) from each single-pair mating were sib-mated in plastic cages (20 cm height × 10 cm diameter) to produce F_2_ offspring. Offspring produced from a single-pair mating was considered as a two-parent family line. The F_2_ screen was conducted in 32-well trays (Advento do Brasil, São Paulo, Brazil). Leaf tissue was excised from leaves of MON 87701 × MON 89788 soybean plants at vegetative stages V4 until V6 and placed in each well. For each insect family line, 128 F_2_ neonates were screened. The number of surviving larvae was recorded and leaf tissue was replaced every four days until pupation. Family lines that presented survivors four days after infestation were retested as described above with additional F_2_ eggs from those lines. Allele frequency was estimated using the algorithm described in equation 1 of Andow and Alstad [39]. Confidence intervals (95% CI) were estimated by equation 5 if no resistant family lines were detected or by equation 7 if resistant family lines were detected. The resistance allele frequency was calculated using the function binom.bayes from the package binom in R 3.1.0.
